# Adaptive immune responses to booster vaccination against yellow fever virus are much reduced compared to those after primary vaccination

**DOI:** 10.1038/s41598-017-00798-1

**Published:** 2017-04-06

**Authors:** Michael Kongsgaard, Maria R. Bassi, Michael Rasmussen, Karsten Skjødt, Søren Thybo, Mette Gabriel, Morten Bagge Hansen, Jan Pravsgaard Christensen, Allan Randrup Thomsen, Soren Buus, Anette Stryhn

**Affiliations:** 1 Department of Immunology and Microbiology, University of Copenhagen, Copenhagen, Denmark; 2 Department of Cancer and Inflammation, Institute for Molecular Medicine, University of Southern Denmark, Odense, Denmark; 3 Department of Infectious Diseases, Copenhagen University Hospital, Copenhagen, Denmark; 4 Medical Office, Copenhagen, Denmark; 5 Department of Clinical Immunology, Copenhagen University Hospital, Copenhagen, Denmark

## Abstract

Outbreaks of Yellow Fever occur regularly in endemic areas of Africa and South America frequently leading to mass vaccination campaigns straining the availability of the attenuated Yellow Fever vaccine, YF-17D. The WHO has recently decided to discontinue regular booster-vaccinations since a single vaccination is deemed to confer life-long immune protection. Here, we have examined humoral (neutralizing antibody) and cellular (CD8 and CD4 T cell) immune responses in primary and booster vaccinees (the latter spanning 8 to 36 years after primary vaccination). After primary vaccination, we observed strong cellular immune responses with T cell activation peaking ≈2 weeks and subsiding to background levels ≈ 4 weeks post-vaccination. The number of antigen-specific CD8+ T cells declined over the following years. In >90% of vaccinees, *in vitro* expandable T cells could still be detected >10 years post-vaccination. Although most vaccinees responded to a booster vaccination, both the humoral and cellular immune responses observed following booster vaccination were strikingly reduced compared to primary responses. This suggests that pre-existing immunity efficiently controls booster inoculums of YF-17D. In a situation with epidemic outbreaks, one could argue that a more efficient use of a limited supply of the vaccine would be to focus on primary vaccinations.

## Introduction

The Yellow Fever virus (YFV) causes acute haemorrhagic fever, which in ≈15% of cases can progress to a more severe, and potentially lethal, stage of the disease^1, 2^. It is a considerable health burden; in the early 1990’es it was estimated that the worldwide annual incidence was 200,000 severe cases and 30,000 deaths; numbers that largely still stands^3^. The virus infects humans that live in, or travel to, parts of tropical and subtropical Africa and South America, where the infection is endemic due to the concurrent existence of transmitting mosquitos and a virus reservoir. The vectors are widespread^4^, and the reservoirs can be found both in humans and non-human primates; conditions that make the disease difficult to control, and virtually impossible to eradicate. Indeed, Yellow Fever re-emerges regularly in endemic areas. The most recent major epidemic outbreak started in Angola in December 2015. As of June 2016, 3,137 suspected cases and 345 deaths have been reported. Further compounding the need for containment and control, this virus is a potential threat to human health in all parts of the world where the transmitting mosquito vectors and the conditions for establishing a reservoir exist e.g. in South-East Asia^1^. In this context, it is worth noting that at least eleven cases of Yellow Fever infected persons traveling from Angola to China have been discovered since December 2015^5, 6^.

In the absence of specific treatment, prevention through vaccination is one of the most effective strategies to reduce the risk of disease and to lower morbidity. The current vaccines against YFV are based on a live attenuated virus strain, YF-17D, which was isolated by Max Theiler and co-workers in 1937^7^ (he was awarded the Nobel prize in Medicine in 1951 for this discovery^8^). Briefly, the pathogenic wild-type Asibi strain was empirically attenuated through multiple adaptations, which involved successive serial passages in Rhesus monkeys, whole mouse embryonic tissue, whole chicken embryonic tissue, and finally denervated chicken embryonic tissue. Over the past 70 years, more than 540 million doses have been administered to humans who live in, or travel to, endemic areas and therefore are at risk of being infected with Yellow Fever virus^9^. The YF-17D vaccine has earned a reputation as one of the most successful vaccines ever developed both in terms of efficacy and safety^10^. This has generated interest in exploring YF-17D as a backbone for chimeric vaccines against other pathogens^11, 12^. It has also generated considerable interest in understanding the nature of the immune responses as well as the mechanisms of protection induced by YF-17D vaccination.

Due to its safety and its nature as a live vaccine, YF-17D vaccination offers a unique model system to study human immune responses during an acute viral infection. In general, antibodies have been considered the dominant effector mechanism responsible for life-long, vaccine-induced immune protection^13–15^. It is now known that many different innate^16–19^ and cellular^16, 20–26^ immune mechanisms, including potent CD4+ and CD8+ T cells responses, contribute to the establishment of long-term immune protection. Here, we recruited 240 healthy volunteers, who were YF-17D vaccinated for travel purposes; 210 were primary and 30 were secondary/tertiary vaccinated. In a prospective, longitudinal cohort study design, we obtained blood donations before and after vaccination. We used these samples to examine and compare the magnitude, quality and dynamics of humoral and cellular immune responses following primary and secondary YFV immunization. In many cases, we used peptide-HLA tetramers to identify and monitor specific T cell responses. Following primary vaccination with the live attenuated virus, we demonstrated a brisk and strong response involving both the humoral and cellular arms of the immune system. Revaccination given 8–36 years after primary vaccination induced much lower responses suggesting that the late memory responses could reduce and possibly control the Yellow Fever virus.

## Materials and Methods

### Approvals, informed consent, animal experiments and accordance

The Danish National Committee on Health Research Ethics approved this study (protocol #H-1-2009-095). The collection of data and cells was approved by The Danish Data Protection Agency (permission #2008-41-2732). All volunteers gave written informed consent prior to participation. Mice were housed in an AAALAC accredited facility in accordance with good animal practice as defined by FELASA. All experiments were performed in accordance with national Danish guidelines including those of the National Animal Ethics Committee (The Animal Experiments Inspectorate).

### Donors and Yellow Fever vaccination

Healthy volunteers, who for traveling purposes were about to be vaccinated against YFV, were recruited at two local vaccination clinics. According to interviews of previous YFV vaccination history, and by examination of each individual’s International Card of Vaccination, these volunteers were assigned as being naïve or previously YFV vaccinated individuals, who were about to receive primary or booster vaccination, respectively. After informed consent had been obtained, the attenuated YFV vaccine, YF-17D-204 (Sanofi Pasteur; marketed as Stamaril in Europe, and as YF-VAX in the USA) was administered intramuscularly. About 42% of the volunteers only received an YFV vaccination, whereas the remaining 58% received additional vaccines. These additional vaccines were typically killed, inactivated or subunit vaccines; in no case was the YFV vaccine co-administered with any other live attenuated vaccines.

### Blood samples and PBMC preparation

Blood samples were obtained before vaccination and at various time points after vaccination (typically day 10–20, range 9 to 41). In some cases, memory samples were collected approximately 2 years after primary vaccination. PBMC from the heparinized blood samples were obtained by Ficoll-Paque density gradient centrifugation and analysed directly, or cryopreserved in 10% DMSO and 90% FCS for later analysis. Sera from the donors were frozen for serological analysis.

### High-resolution HLA-typing

Chromosomal DNA was isolated from the PBMC’s and used to perform high-resolution (i.e. 4 digit) HLA-typing (Genome Diagnostics, Utrecht, The Netherlands). All loci encoding classical (i.e. polymorphic, peptide presenting) HLA molecules were identified. This included three class I Ioci, HLA-A, -B, -C and six class II loci, HLA-DRB1, -DRB3,4,5, -DQA1, -DQB1, -DPA1 and -DPB1.

### Activation marker analysis

Initially, we analysed the complete CD8^+^ T cell activation phenotype, CD38^+^, HLA-DR^+^, Ki-67^+^ and Bcl-2^low^, suggested by Miller *et al*.^26^. However, after having analysed the first approximately 190 donor samples (Supplementary Figure S1), we came to the conclusion that we would lose very little information in terms of the size of the activated CD8^+^ T cell population if we only used the two extracellular markers, rather than the complete activation phenotype.

Thus, PBMCs were analysed *ex vivo* for T cell (CD3, CD4, CD8) and the extracellular T cell activation markers, CD38, HLA-DR. Briefly, PBMCs were incubated with fluorochrome-conjugated anti-CD3, -CD4, -CD8, -CD38 and -HLA-DR antibodies for 30 minutes at room temperature, washed, fixed with 1% formaldehyde, and analysed by flow cytometry (LSR-II, BD Biosciences) using Diva software. For representative FACS plots see Supplementary Figure S2.

### Peptides and antibodies

A systematic library of overlapping 15-mer peptides spanning the entire YF-17D proteome (UniProt# P03314) of 3411 amino acids was synthesized (all peptides were supplied HPLC-purified (minimum 80%, in most cases >95%), mass spectrometry validated and lyophilized by Schafer-N (Copenhagen, Denmark)). Each peptide overlapped its neighbouring peptides with 11 amino acids. The approximately 850 peptides (the synthesis of a few peptide failed) were organized into 30 pools with 28–30 peptides in each pool. The resulting peptide pools were used to stimulate YFV-specific T cell responses.

All monoclonal antibodies were obtained from BioLegend (San Diego, CA, USA), except for the previously reported anti-YF NS3 monoclonal antibody, 12-25-6^27^, which was purified from culture supernatants.

### Cell culture and peptide stimulation

PBMCs were incubated over night with a mixture of relevant peptides at a concentration of 0.5 µM of each peptide in X-vivo 15 (Fisher Scientific) media supplemented with 5% AB Serum (Invitrogen). The cells were subsequently harvested, washed and plated at a concentration of 5 × 10^6^/ml supplemented with 50U/ml IL-2 for expansion. Fresh media and IL-2 were supplemented every second day until the cells were harvested at day 8, and IL-15 (15ng/ml) was added the last four days.

### Tetramers

HLA class I tetramers were produced as previously described^28^. Briefly, recombinant, biotinylated HLA class I heavy chain, human β_2_-microglobulin and peptide were incubated in 50 mM tris-maleate pH 6.6 and 0.1% Pluronic F68 for 48 h at 18 °C. The resulting monomers were tetramerized by addition of fluorochrome labelled Streptavidin (Streptavidin-PE or Streptavidin-APC; all from BioLegend) sequentially over 60 min at a 1:4 molar ratio of streptavidin to monomer. Here, we have used tetramers representing 12 YFV epitopes. Three have previously been published: NS4B_214-222_ (LLWNGPMAV)-A*02:01^21^; NS5_672-680_ (RPIDDRFGL)-B*07:02^29^; NS2A_4-13_ (HAVPFGLVSM)-B*35:01^23^. Nine are novel epitopes: NS5_286–295_ (KSEYMTSWFY)-A*01:01; NS3_4-12_ (VLWDIPTPK)-A*03:01; NS3_24-32_(IYGIFQSTF)-A*24:01; NS5_476-484_ (WYMWLGARY)-A*29:02; NS1_116-124_ (KTWGKNLVF)-A*32:01; EnvE_207–215_ (RQWAQDLTL)-B*13:02; NS3_73-81_ (SVKEDLVAY)-B*15:01; EnvE_200-209_ (TESWIVDRQW)-B*44:02; NS3_207-213_ (TRRFLPQIL)-C*06:02. The identification of these epitopes will be described elsewhere.

Pellets of 10^6^ PBMCs obtained *ex vivo*, or pellets from 2 ×10^5^ cells obtained from *in vitro* peptide stimulated cell cultures, were re-suspended in a 10 µl tetramer solution at a final concentration of ≈30 nM, and incubated for 20 min at room temperature, followed by 30 min incubation with fluorochrome conjugated anti-CD3, -CD8, -CD38 and -HLA-DR antibodies. The cells were analysed by flowcytometry (Fortessa or LSR-II, BD Biosciences) using Diva software. Supplementary Figure S3, a NS4B_214-222_-A*02:01 tetramer *ex vivo* staining pre- and post-YF vaccination, illustrates the tetramer background level.

### *Ex-vivo* Interferon-γ ELI Spot assay

As previously described, fresh or thawed PBMCs were tested using an Interferon-γ specific ELI Spot assay^30^. Briefly, 2–3 × 10^5^ cells/well were plated in an ELI Spot plate (MAHAS4510, Merck Millipore, USA) and *in vitro* cultured for 18–24 hours in media supplemented with or without peptide at 0.5 µM (or, as positive control, with 1 µg/ml Staphylococcal enterotoxin B (SEB, Sigma Aldrich, St. Louis, USA). AP conjugate substrate kit (Bio-Rad) was used for visualization of spots. ELI spot were counted using a CTL ImmunoSpot series 5 UV Analyzer and ImmunoSpot 5.0.9 software (C.T.L., Shaker Heights, USA) was used for analysis. Wells with SPU >2 times the background wells were considered positive.

### YF-17D virus preparation and quantitation

YF-17D virus (Stamaril, Sanofi Pasteur; reconstituted as recommended by the manufacturer) was propagated in Vero (ATCC CCL-81) cells as previously described^27^. In brief, cell monolayers were seeded 24 hours earlier and infected with at a multiplicity of infection (MOI) of 0.001/cell in DMEM 2% FCS. Infectious supernatants were harvested when cytopathic effect was more than 60% (day 6 p.i.), freeze-thawed and clarified of cell debris at 2000 rpm for 15 min at 4 °C. Viral stocks were stored as single use aliquots at −80 °C.

Infectious virus titres were determined by an Immuno Focus Assay (IFA) as previously described^27^. In brief, virus stocks were serially diluted (ten-fold) in DMEM and adsorbed for 1 h at 37 °C onto Vero (ATCC CCL-81) cell monolayers in twenty-four-well plates; cells were then overlaid with media containing 0.9% methyl cellulose and incubated at 37 °C for 3 days. Following fixation and permeabilisation, a monoclonal antibody, 12-25-6^27^, directed against NS3 from YF-17D virus was used for detection of virus antigens within infected cells; the foci of infection (analogous to plaques) were visualized using HRP-substrate reaction and counted.

### Plaque reduction neutralization test (PRNT) and titre

Appropriately diluted YF-17D (generating 70–100 pfu/well) was incubated 1 h at 37 °C with serial 2-fold dilutions of pre- or post-vaccination serum from primary or booster-vaccinated donors. The resulting virus-serum mixtures were added to monolayers of Vero cells in 24-well plates, incubated at 37 °C for 2 h, overlaid with media containing 0.9% methyl cellulose, and the incubation continued for another 3 d at 37 °C. After fixation (4% formaldehyde) and permeabilisation (0.5% Triton-X100 in Hanks media), a monoclonal mouse antibody, 12-25-6, specific for the N-terminal end of NS3 from the YF-17D virus was used for detection of infected cells. Foci of infection were visualized with an HRP-conjugated secondary antibody, and subsequently counted. The PRNT_50_ titre was defined as the reciprocal of the last serum dilution, which caused a 50% reduction in the number of plaques. All PRNT values were log transformed before statistical analysis.

### Recombinant adenoviral vectors

A recombinant Adenovirus 5 (Ad5) vector encoding the structural proteins core, membrane and envelope (C, M, E) from YF-17D virus (GenBank# X03700.1) was produced through homologous recombination by standard methods^31, 32^. After purification on a caesium chloride (CsCl) density gradient, the viral stock was immediately frozen as single use aliquots in 10% glycerol at −80 °C, and the infectivity of the stock was determined using the Adeno-X Rapid Titer kit (BD Clontech). The presence of the inserted transgenes in the recombinant Ad5 vector was validated by sequence analysis prior to its use for vaccination in mice.

### Mice

Female C57BL/6 (wt. B6) mice were obtained from Taconic Farms and housed in a pathogen – free facility; animals were used in experiments when 7–10 weeks old. All experiments were approved by the national animal ethics committee and performed in accordance to the national guidelines.

### Vaccination of mice

For adenovirus vaccination, 2 × 10^7^ pfu of recombinant Ad-5 in a volume of 30 µl was given subcutaneously to isofluorane-anesthetized mice in the right hind footpad. Vaccination with YF-17D virus at the dose of 10^5^ pfu in 300 µl was given subcutaneously (s.c.) at the base of the tail.

### Spleen cell preparation and flow cytometry

Single cell suspensions of splenocytes were obtained by pressing the organs through a fine steel mesh (mesh size, 70 µm), followed by centrifugation and re-suspension in RPMI 1640 cell culture medium. The frequencies of antigen-specific CD8^+^ T cells in the spleen were determined by intracellular cytokine staining (ICS) performed after 5 h of incubation with relevant peptides (0.1 µg/ml of NS3 _(268-275)_ or E _(4-12)_) in the presence of monensin (3 µM) at 37 °C in 5% CO_2_. After incubation, the cells were stained with Abs for cell surface markers (peridinin chlorophyll protein-Cy5.5-CD8, APC-Cy7-CD44) and Ab for intracellular cytokine (APC- interferon gamma (IFN-γ)). Samples were run on an LSRII flow cytometer (BD biosciences) and analysed using FlowJo software (TreeStar).

### Statistics

GraphPad Prism (version 5) was used for statistical analyses (unpaired and paired Mann-Whitney-Wilcoxon tests, unpaired and paired t-tests, Fishers exact test, and ROC analysis).

To establish a background frequency of activated CD38+HLA-DR+CD8+ T cells, we examined the data from blood samples taken before primary or booster vaccinations. A few donors had high frequencies of activated CD8+ T cells in the blood samples taken before vaccination; something that could be caused by on-going immune responses against unrecognized infections. These outliers were excluded using “Iglewicz and Hoaglin’s robust test for multiple outliers” (two-sided test, with the recommended setting of Z = 3.5, http://contchart.com/outliers.aspx). Statistical analyses of samples taken from the remaining donors before vaccination were used to calculate the background frequencies of activated CD4+ or CD8+ T cells. As expected if outliers were caused by on-going immune responses, being a CD4+ outlier was significantly correlated with being a CD8+ outlier (two-tailed P value of 0.0014, Fishers exact test). Exclusion of outliers was only performed to establish background frequencies; it was not for used in the context of group comparisons.

## Results

### Recruiting volunteers

Vaccination with the live, attenuated Yellow Fever vaccine, YF-17D, is an ideal opportunity to investigate the dynamics and quality of human immune responses following exposure to an acute primary virus challenge under safe and highly controlled conditions. Volunteers, who for travel purposes were about to receive YF-17D vaccination, were examined. A 50 ml blood donation was obtained immediately before vaccination, and a 200 ml blood donation was obtained about 10–20 days after vaccination. During the two-year period of 2010 to 2011, a total of 294 volunteers were recruited (65% were female and 35% were male; matching the gender distribution of Danish blood bank donors). We obtained donations before and after vaccination from 249 (85%) of the volunteers (the remaining 45 volunteers did not give after vaccination donations). Subsequently, nine volunteers were excluded (due to viral hepatitis infection, low PBMC yields, or insufficient information about prior immunization). Thus, paired (i.e. before and after vaccination) blood donations from 240 volunteers were available for these studies: 210 primary, 28 secondary and two tertiary vaccinees. The age range for the primary vaccinated group was 18–68 years, with an average age of 28.4 years; and the age range of the secondary/tertiary vaccinated group was 21–61 years, with an average age of 38.5 years. For twelve of the primary vaccinees, an additional 200 ml blood donation was obtained approximately 2 years after vaccination. Thus, we obtained blood at five different time points yielding T cells at different stages of development denoted: (1) **naïve T cells**, which were sampled just before primary vaccination, (2) **primary effector T cells** sampled after primary vaccination, (3) **primary memory T cells** sampled about 2 years after primary vaccination, (4) **late memory T cells** sampled just before booster vaccination, which typically occurs about 10 years, or more, after primary vaccination and finally (5) **secondary effector T cells** sampled after booster vaccination. In addition, the responses of two primary vaccinees were followed longitudinally using multiple smaller blood donations spaced over a longer time period.

### Background frequency of activated CD8+ T cells

**Figure 1:**
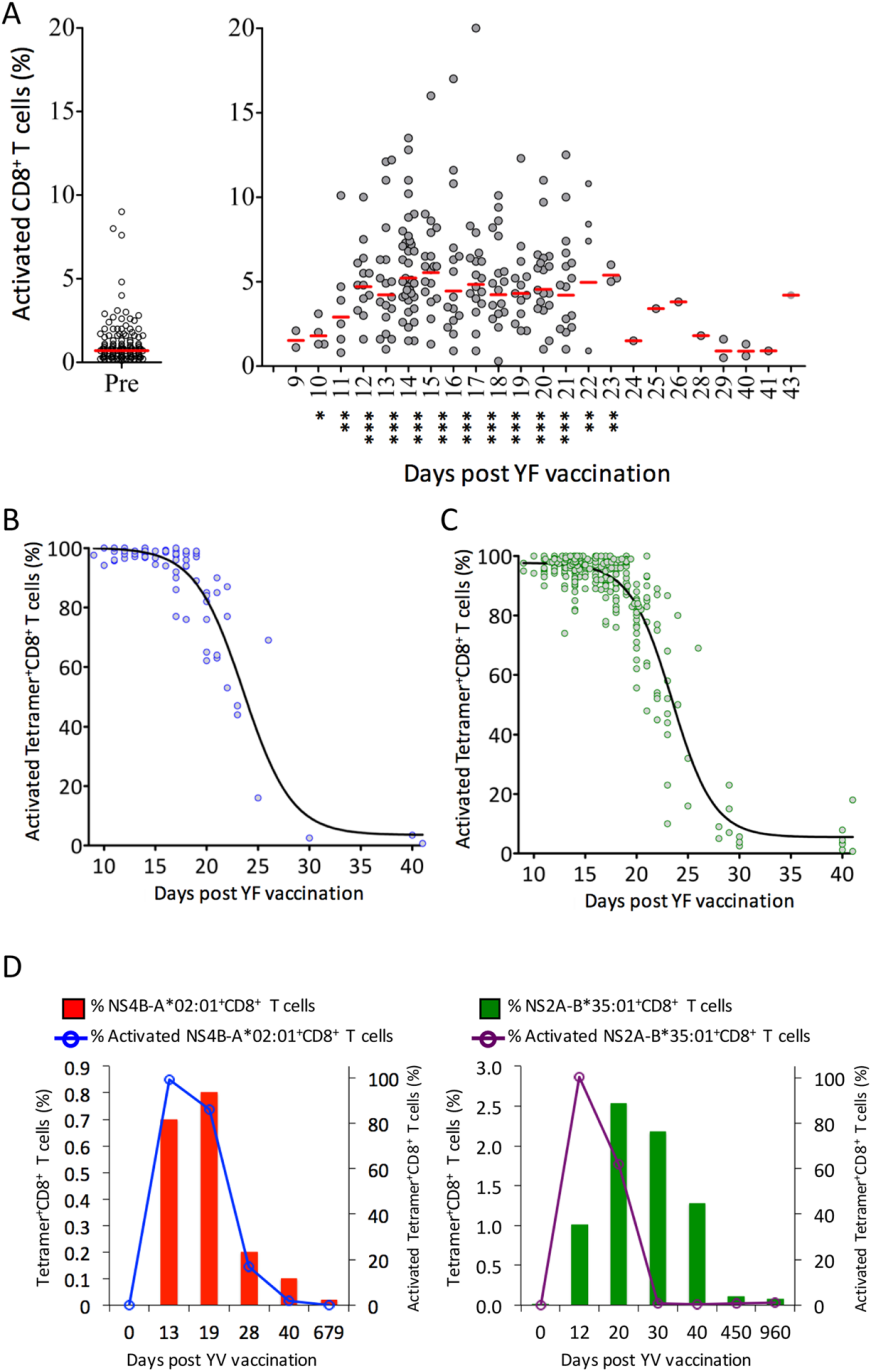
Activation of CD8+ T cells after primary YF-17D vaccination. PBMCs were analysed by flow cytometry. Activated CD8^+^ T cells were defined as CD38^+^ HLA-DR^+^ CD8^+^ CD3^+^ T cells (see supplementary Figure S1, left-hand panel). (**A**) Frequencies of activated CD8^+^ T cells before and after YFV vaccination. Pre- (day 0) and post-vaccination (day 9 to 43) PBMCs from 209 primary YFV vaccinated donors were analysed. In the left-hand panel, pre-vaccination frequencies are plotted; in the right-hand panel, the post-vaccination frequencies are plotted according to the day the blood sample was collected. The median for each collection day is indicated. Mann Whitney U test was used to determine the significance of the difference between day 0 pre-vaccination, and the indicated day post vaccination (***p < 0.001; **p < 0.01; *p < 0.05). (**B**) The frequencies of activated and NS4B_214-222_/HLA-A*02:01 tetramer specific CD8+ T cells from 93 HLA-A*02:01 donors were analysed and plotted according to the day the blood samples were collected. (**C**) The previous analysis was extended to 11 different HLA- class I tetramer-specific CD8+ T cells from 41 primary vaccinated donors yielding 321 measurements of frequencies of activated and HLA class I tetramer positive CD8^+^ T cells, which are plotted according to the day the blood sample was collected. (**D**) PBMCs from two primary vaccinated donors were collected at various time points before and after primary YFV vaccination. In the left-hand panel, the frequencies of activated and NS4B_214-222_/HLA-A*02:01 tetramer positive CD8^+^ T cells from donor YF4967 are plotted according to the day the blood samples were collected. In the right-hand panel, the frequencies of activated and NS2A_4-13_/HLA-B*35:01tetramer positive CD8^+^ T cells from donor YF7512 are plotted according to the day the blood samples were collected. The frequencies of tetramer^+^ CD8^+^ T cells are depicted as histograms with values given on the left Y-axis; whereas the frequencies of activated tetramer positive CD8^+^ T cells are depicted as a line plot with values given on the right Y-axis.

To establish a background frequency of activated CD8 + T cells, we examined the frequency of CD3+, CD8+, CD38+, HLA-DR+ T cells in PBMC’s sampled before vaccination. Removing outliers, the background frequency of activated CD8^+^ T cells was found to be 0.87% (N = 163, SD = 0.59 with a 99% confidence interval of the mean from 0.75% to 0.99% (Fig. 1A). A background activation frequency level of about 1% corresponds well to those observed in other studies^26, 29, 33^. About 6% of the donors had apparent background frequency of 3% to almost 14% activated CD8+ T cells, and were removed as outliers. We speculate that these donors at the time of sampling might have had unnoticed infectious or inflammatory conditions.

### Rapidly expanding and contracting primary effector CD8+ T cell responses

We adopted a longitudinal study design for our study. Each donor was asked to give a blood sample approximately 14 days after vaccination, however, the donor themselves chose the exact time based on their convenience. These post-vaccination donations were obtained on average 16.4 days (SD = 3.9 days) with a range from 9 to 41 days after vaccination. Data obtained from 209 of 210 primary vaccinees was used to establish the kinetics of the CD8^+^ T cell activation response. The frequency of CD8^+^ T cells being of the CD38+, HLA-DR+ activated phenotype increased from day 9–10 after vaccination. Around day 14–15, it reached a plateau with an average of 5–7%, however, covering a wide range from 2% to 20%. This plateau lasted for about a week, and at day 22–23, the frequency of activated CD8^+^ T cells contracted and reached baseline level around day 29 (Fig. 1A). Thus, the acute virus infection caused by YF-17D vaccination led to expansion (Fig. 1D) and activation (Fig. 1A) of CD8^+^ T cells entailing a response cycle of about four weeks; two weeks of expansion, a plateau period of about a week with a high level of activated T cells in the peripheral blood, and finally a contraction period of about a week.

Next, we used peptide-HLA class I tetramers to examine the kinetics of activation of antigen-specific CD8^+^ T cells induced by primary YF-17D vaccination. Initially, we used a tetramer representing the HLA-A*02:01-restricted NS4B_214-222_ epitope identified by Akondy *et al*.^21^. This is a particularly feasible specificity to monitor since the HLA-A*02:01 allotype is the most frequent HLA-I allele in our donors, and the NS4B_214-222_ epitope is an unusually strong immunogen in the context of HLA-A*02:01. To examine the CD8^+^ activation kinetics, we examined NS4B_214-222_ specific, HLA-A*02:01-restricted CD8^+^ T cell responses in 93 HLA-A*02:01 donors. This immunodominant epitope was very frequently recognized (in fact, every single of the 93 primary YF-17D vaccinated, HLA-A*02:01-positive individuals examined responded to this epitope) and it led to very strong responses (up to 11% of all CD8^+^ T cells recognized this epitope at the peak of the primary effector response). Therefore, monitoring this specific response is a unique opportunity to examine the kinetics of activation and deactivation *ex vivo* during a primary YF-17D vaccination response (Fig. 1B). Early in the response, from the earliest point of observation at 9 days and until day 16 after vaccination (in some donors even up to day 19), 90–100% of the tetramer positive CD8^+^ T cells were of the activated phenotype. Subsequently, a sharp decline in the level of activated, NS4B_214-222_specific, HLA-A*02:01-restricted CD8+ T cells, was observed starting at day 19 and returning to baseline levels around day 30 (Fig. 1B).

Considering that the NS4B_214-222_-specific, HLA-A*02:01 response is unusually immunodominant, it could have unusual kinetics. We therefore extended the kinetics analysis of CD8^+^ primary effector T cell activation to a larger group of YF-17D-specific CD8^+^ T cell responses identified in primary vaccinated donor cohort (Fig. 1C). Using a panel of 11 different YF peptide-HLA-I tetramers (Supplementary Figure S4 shows representative stainings of these 11 tetramers, which are also given in the tetramer section of Materials and Mathods; a detailed description of the identification of these epitopes will be described elsewhere), we analysed the frequency of activation of YF-17D-specific CD8^+^ T cells from 41 primary vaccinated donors. This led to the generation of 321 data points. The early development of almost fully activated CD8^+^ T cells followed by a sharp decline in activation around day 20 reaching baseline around day 30 is completely superimposable with the NS4B_214-222_-specific, HLA-A*02:01 response. Thus, this activation kinetic seems to be very consistent across many different CD8^+^ T cell specificities directed against this attenuated virus.

Finally, we used tetramers to monitor both CD8^+^ T cell expansion and activation in two donors where the frequencies and activation status of two known immunodominant CD8+ T cell responses directed against the HLA-A*02:01-restricted NS4B_214-222_ epitope^21^ and the HLA-B*35:01-restricted NS2A_4-13_ epitope^34^, respectively, were examined at several consecutive time points after vaccination (Fig. 1D). The tetramer specific CD8^+^ T cells expanded rapidly and peaked about 3 weeks post vaccination. At the peak of the response, as much as 2.5% and 0.85% of the CD8^+^ T cell populations were dedicated to the recognition of these two immunodominant epitopes, respectively. Subsequently, the specific CD8^+^ T cell populations contracted rapidly over the course of 2–3 weeks. Over the following years, the frequency slowly declined; at day 450 and 960 of the NS4B_214-222_-specific, HLA-A*02:01-restricted response, the tetramer positive cells accounted for 0.11% and 0.08%, respectively, of the entire peripheral CD8^+^ T cell population, and at day 679 of the NS2A_4-13_-specific, HLA-B*35:01-restricted response, the tetramer positive cells accounted for 0.02%. Co-staining the tetramer positive CD8+ T cells with CD38, allowed us to determine the activation status of the tetramer positive CD8+ T cells during these responses. In agreement with the activation kinetics observed in Fig. 1, all tetramer positive CD8^+^ T cells were activated at day 12, this had dropped to ≈70% at day 20, and further dropped to less than 17% at day 30, and ≈0% at day 40. Thus, during CD8+ T cell contraction, an increasing fraction of the dwindling population of specific CD8^+^ T cells becomes inactive (at least as defined by CD38 expression). This leaves a transitory population of specific, yet inactive, CD8^+^ T cells appearing sometime between day 12 and day 20 after vaccination and eventually accounting for almost all the specific CD8^+^ T cells observed after day 30. This is in agreement with a transition occurring of effector to memory T cells as described by Miller *et al*.^26^.

### CD8+ T cell responses of booster-vaccinated donors are reduced compared to primary vaccinated donors

**Figure 2:**
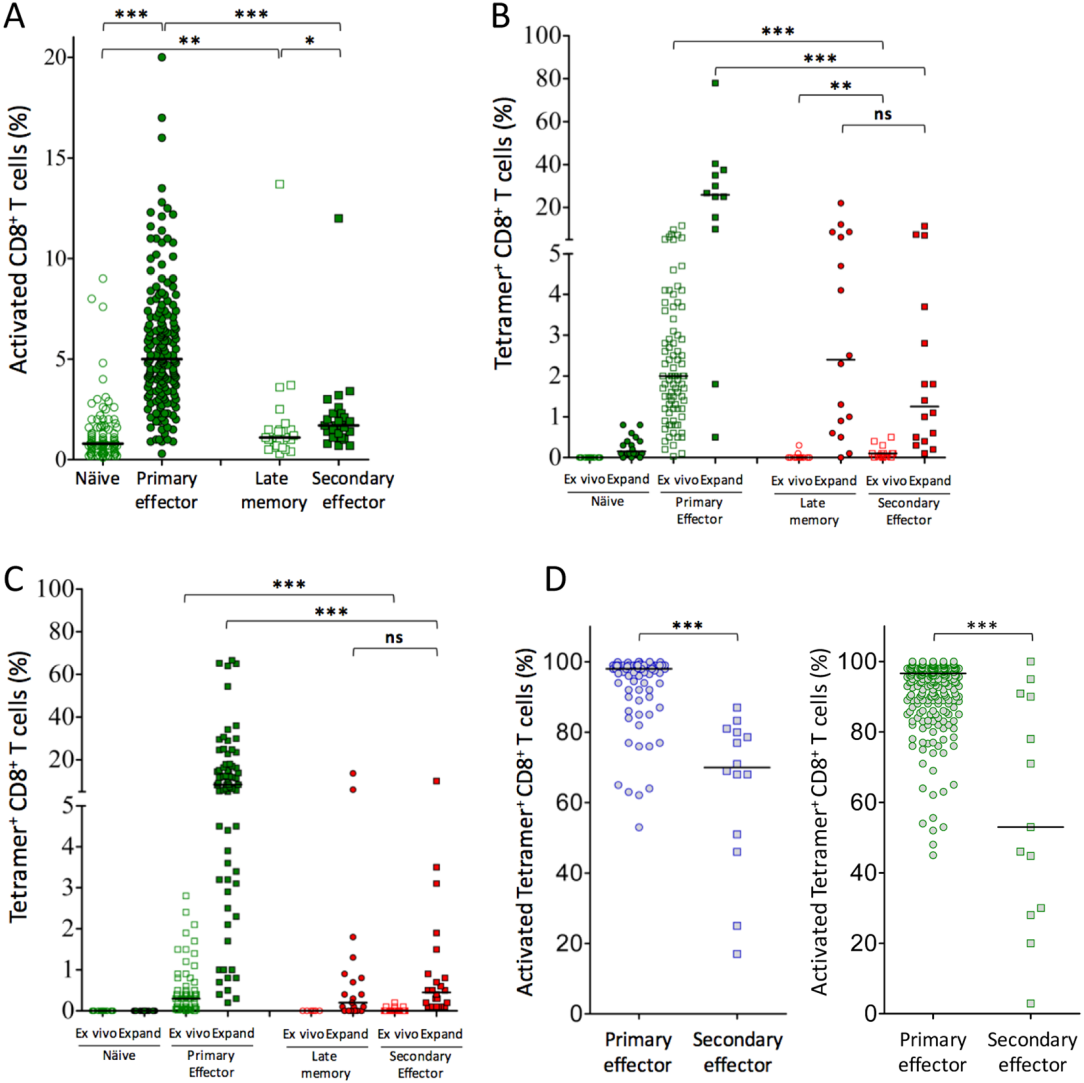
CD8^+^ T cell responses in primary and booster vaccinated donors. PBMC’s were obtained from primary and booster vaccinated donors either before (day 0) or at the peak of the vaccine-induced response (day 12–22). (**A**) The frequencies of activated CD8^+^ T cells (i.e. CD38^+^ HLA-DR^+^ CD8^+^ CD3^+^ T cells) obtained before (denoted “naïve”; n = 127) and after (“primary effectors”; n = 180) primary YFV vaccination as well as before (“late memory”; n = 26) and after (“secondary effectors”; n = 26) booster vaccination. The median is indicated. Mann Whitney U test was used to determine the significance of the difference between the indicated groups (***p < 0.0001; **p = 0.009; *p = 0.013). (**B**) PBMC’s obtained from donors before (“naïve”) and after (“primary effectors”) primary vaccination, and from donors before (“late memory”) and after (“secondary effectors”) booster YFV vaccination were enumerated for NS4B_214–222_-specific, HLA-A*02:01-restricted CD8^+^ T cells. These analyses were performed both *ex vivo* (denoted “*ex vivo*”) and after an 8-day *in vitro* peptide stimulation and expansion culture (denoted “Expand”). Results are given as frequencies of tetramer^+^ CD8^+^ T cells. Mann Whitney U test was used to determine the significance of the difference between the indicated groups (***p < 0.0001; **p = 0.002; ns: not significant). (**C**) The experiments in B were repeated using a panel of 11 different YFV-derived/HLA class I tetramers. Mann Whitney U test was used to determine the significance of the difference between the indicated groups (***p < 0.0001; ns: not significant). (**D**) The activation status of YFV-specific CD8^+^ T cell was determined post vaccination for donor samples collected between day 12 and day 22 after YFV vaccination. In the left-hand panel, PBMCs from primary vaccinated donors (“primary effectors”; n = 79) and from booster vaccinated donors (“secondary effectors”; n = 14) were stained *ex vivo* with a NS4B_214-222_/HLA-A*02:01 tetramer. In the right-hand panel, PBMCs from primary vaccinated donors (“primary effectors”; n = 279) and from booster vaccinated donors (“secondary effectors”; n = 13) were stained *ex vivo* with tetramers relevant for their HLA haplotype. The activation status of these tetramer positive CD8+ T cells was determined as above. The median is indicated. A Mann Whitney U test was used to determine the significance of the difference between the groups (***p < 0.0001).

Our study included 30 healthy volunteers, who received booster YF-17D vaccinations; 26 of these were sampled 12 to 22 days after vaccination, the time period of the activation plateau with consistent responses across all donors. The time between the primary and the booster vaccination ranged from 8 to 36 years. CD8^+^ T cells sampled from the boosted donors were examined for the expression of activation markers CD38 and HLA-DR before and after the booster vaccination (Fig. 2A). Although we did observe a significant, albeit modest, ≈0.5% increase in the number of activated CD8+ T cells after booster vaccination, this increase is much smaller than the ≈4% increase we observed after primary vaccination (Fig. 2A). In other words, the absolute activation levels achieved after primary vaccination were much higher than after booster vaccination, and this difference was highly significant (Fig. 2A).

To further analyse the difference between primary and boosted donors at the peak of the response from 12 to 22 days after vaccination, we selected HLA-A*02:01 positive donors from both the primary and booster vaccinated cohorts, and used the NS4B_214-222_/HLA-A*02:01 tetramer (described above) to enumerate the *ex vivo* frequency of corresponding CD8^+^ T cells before and after vaccination in both groups (Fig. 2B). For the primary vaccinated donors, NS4B_214-222_/HLA-A*02:01 tetramer-specific CD8^+^ T cells were absent in the naïve T cell samples, but were observed in all 85 primary effector samples examined (frequencies from 0.1 to 11.5% of CD8^+^ T cells). For the booster-vaccinated donors, NS4B_214-222_/HLA-A*02:01 tetramer-specific CD8^+^ T cells were observed in only 2 of the 12 donors examined before booster vaccination (reaching frequencies of only about 0.3% at this late memory stage), and in 14 of the 15 donors examined after booster vaccination (reaching very low frequencies; only three donors had frequencies >0.1% at this secondary effector stage).

These scarce and weak *ex vivo* late memory responses made us examine whether NS4B_214-222_/HLA-A*02:01 tetramer-specific CD8^+^ T cells could be detected after *in vitro* culture with the NS4B_214-222_ peptide for 8 days (Fig. 2B, which shows naïve, primary effector, late memory and secondary effector responses *ex vivo* and after *in vitro* culture). Indeed, after *in vitro* culture, NS4B_214-222_-specific, HLA-A*02:01-restricted CD8^+^ T cells could be identified in 15 of the 16 late memory cultures, and in all primary and secondary effector cultures. However, we also observed clearly detectable, albeit much lower, NS4B_214-222_-specific responses in 15 of 18 naïve cultures suggesting that NS4B_214-222_-specific effector and memory responses observed after *in vitro* culture may not necessarily be caused by vaccination. Noting that the HLA-A*02:01-restricted NS4B_214-222_ epitope is unusually strong and immunodominant, we again turned to the panel of 11 tetramers representing other immunodominant YF-17D-specific, HLA class I restricted CD8^+^ T cell epitopes (Fig. 2C). For the *ex vivo* responses, we found the same picture as with the HLA-A*02:01-restricted NS4B_214-222_ epitope: no naive responses, strong and frequent primary effector responses, absent late memory responses, and an even weaker and less frequent secondary effector responses (six detectable responses in 23 donors analysed). For the *in vitro* peptide stimulation responses, unlike for the NS4B_214-222_-specific, HLA-A*02:01-restricted CD8^+^ T cell responses, we did not observe any *in vitro* stimulation of cells from naïve individuals; something, which allowed us to interpret with greater certainty the *in vitro* experiments with CD8^+^ T cells obtained before or after a booster vaccination. In general, specific CD8^+^ T cells can only be *in vitro* expanded in vaccinated individuals. By this token, we found recruitable late memory YF-17D-specific T cells in the blood 10–30 years after vaccination. Strikingly, we observed strong *ex vivo* expansions of CD8+ T cells after primary vaccination; expansions that were much stronger than after booster vaccination.

Finally, we examined the degree of activation as determined by CD38^+^ and HLA-DR^+^ expression on YF-17D-specific, HLA class I tetramer-specific CD8^+^ T cells in primary and boosted donors. To this end we analysed blood samples, which had been donated between day 12 and day 22 after vaccination, where the activation level was at peak plateau level in primary vaccinated. In 66 of 79 HLA-A*02:01 positive and primary vaccinated donors examined, 85–100% of the NS4B_214-222_/HLA-A*02:01 tetramer positive CD8^+^ T cells were CD38^+^, whereas the corresponding figure for boosted donors tended to be less than 75% (Fig. 2D, left-hand panel). That this is a general phenomenon was further demonstrated when other YF-17D specific CD8^+^ T cells were examined (Fig. 2D, right-hand panel). Altogether, the CD8^+^ T cell response was significantly reduced following booster vaccination compared to primary vaccination, and in many cases, virtually absent.

### CD38+, CD4+ T cell responses after primary and booster vaccination

Using the same approach as for the CD8 + T cells, we established a background frequency of activated CD4^+^, CD38^+^, HLA-DR^+^ T cells. It was found to be 0.35% (N = 159, SD = 0,166 with a 99% confidence interval of the mean from 0.32% to 0.39%. About 4% of the donors had apparent background frequency of 0.9% to almost 11% activated CD4^+^ T cells, and were removed as outliers. As for CD8^+^, CD38^+^, HLA-DR^+^ outliers, we speculate that these activated CD4^+^ outlier donors at the time of sampling might have had unnoticed infectious or inflammatory conditions.

**Figure 3:**
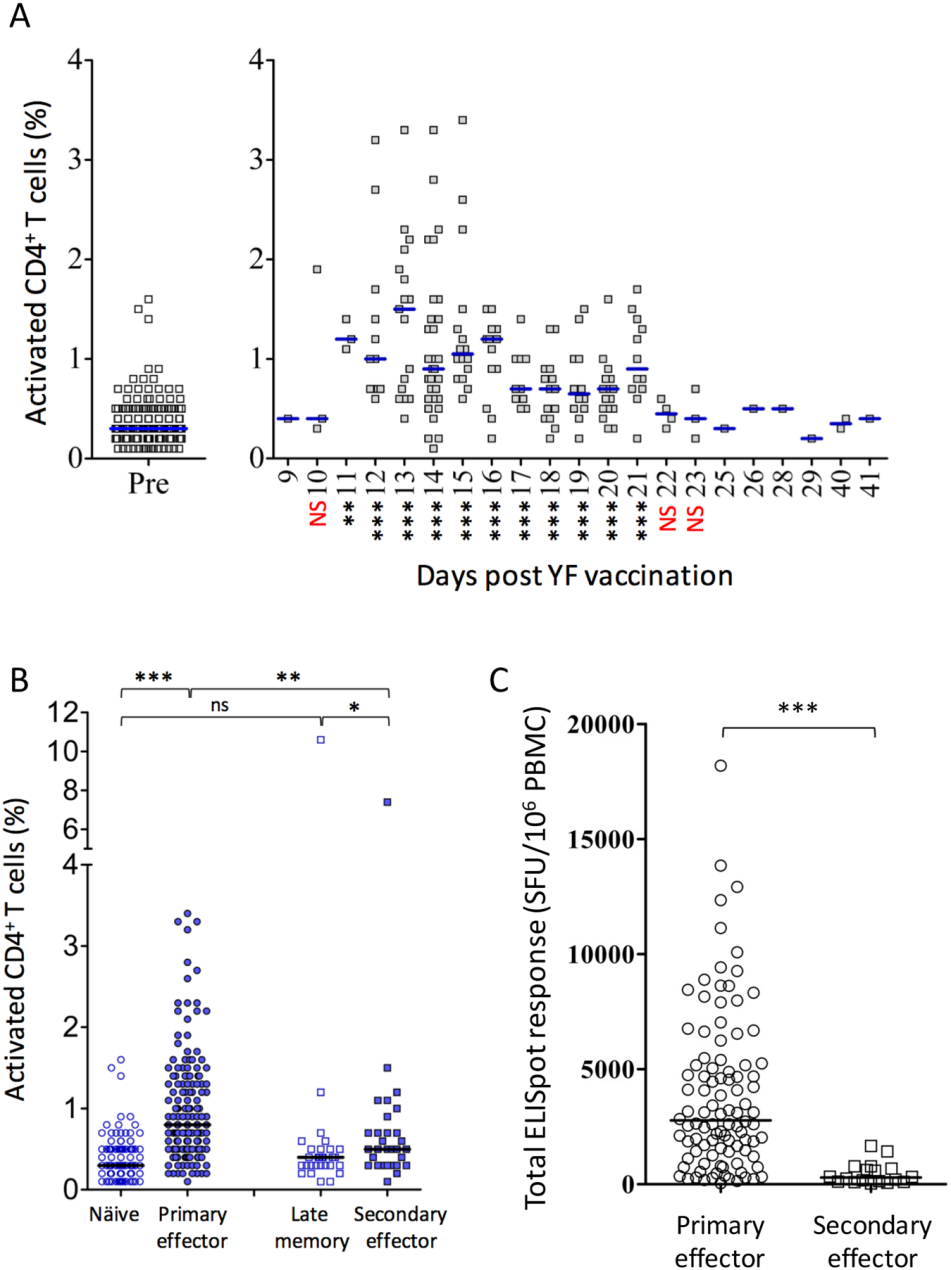
Activation of CD4+ T cells after primary and booster YF-17D vaccination. PBMCs were analysed by flow cytometry, and activated CD4^+^ T cells were defined as CD38^+^ HLA-DR^+^ CD4^+^ CD3^+^ T cells (see supplementary Figure S2, right-hand panel). (**A**) Frequencies of activated CD4^+^ T cells before and after YFV vaccination. Pre- (day 0) and post-vaccination (day 9 to 41) PBMCs from 209 primary YFV vaccinated donors were analysed. In the left-hand panel, pre-vaccination frequencies are plotted; in the right-hand panel, the post-vaccination frequencies are plotted according to the day the blood sample was collected. The median for each collection day is indicated. Mann Whitney U test was used to determine the significance of the difference between day 0 pre-vaccination, and the indicated day post vaccination (***p < 0.001; **p = 0.004; if nothing is given the sample size is too small). (**B**) The activation level of CD4^+^ T cells from primary and booster vaccinated donors before and after YFV vaccination. CD4^+^ T cell activation status was determined for PBMCs from primary vaccinated donors before (“naïve”; n = 147) and after (“primary effectors”; n = 178) vaccination and for PBMCs from booster vaccinated donors before (“late memory”; n = 29) and after (“secondary effectors”; n = 29) vaccination. The median is indicated. A Mann Whitney U test was used to determine the significance of the difference between the indicated groups (***p < 0.0001; **p = 0.001; *p = 0.0264; ns: not significant). (**C**) The total YFV specific CD4+ and CD8+ T cell responses in primary and booster vaccinated donors were analysed. PBMCs from primary vaccinated donors (“primary effector”; n = 100) and from booster-vaccinated donors (“secondary effector”; n = 18) were analysed *ex vivo* using IFN-γ specific ELISpot analysis using 30 peptide pools covering the entire YFV proteome. Only donors vaccinated 12–22 days prior to blood collection were included. For each donor, the total YFV specific T cell response was measured as the sum of IFN-γ specific cells (spot forming units, SFU per 10^6^ PBMC). The median is indicated. Mann Whitney U test was used to determine the significance of the difference between the groups (***p < 0.0001).

We then examined the expression level and kinetics of the CD38 activation marker on CD4^+^ T cells during the acute immune response to YF-17D vaccination. Whereas up to 15–20% of CD8^+^ T cells expressed CD38 at the peak of the CD8^+^ T cell response, only up to 3–4% of CD4^+^ T cells expressed CD38 at the peak of the CD4^+^ T cell response (Fig. 3A). Similar to the CD8^+^ T cell response, the CD38 expression kinetics on CD4^+^ T cells rose rapidly around day 9 and peaked around day 14. The contraction phase started slightly earlier than for CD8^+^ T cells and reached a 0.5% level at day 22 (Fig. 3A). The expression levels of CD38 activation markers on CD4^+^ T cells after primary vaccination revealed a highly significant response (i.e. comparing naïve vs. primary effector samples, P < 0.0001), whereas no response was observed after booster vaccination (Fig. 3B, P < 0.17). Thus, like the CD8^+^ T cell response, the CD4^+^ T cell response was significantly reduced following booster vaccination compared to primary vaccination, and in many cases virtually absent.

### Functional T cell responses after primary and booster vaccination as measured by IFN-γ ELI Spot

To assess the functional cellular immune responses in primary and boosted donors and compare them quantitatively, we used PBMCs obtained from donors after vaccination and performed *ex vivo* IFN-γ ELI Spot analysis. Note, that this analysis compounds CD4^+^ and CD8^+^ T cell responses. For each of 100 primary and 18 booster-vaccinated donors, we analysed a total of 30 pools each containing up to 30 overlapping peptides (15 amino acids in length with an overlap of 11 amino acids) spanning the entire YF-17D proteome. Thus, summing up the number of IFN-γ producing cells per 10^6^ cells obtained for all 30 peptide pools for a given donor gives a measure of the total anti-YF-17D T cell response of that donor (Fig. 3C). Albeit responses were detected in both primary and secondary effector T cells (i.e. after primary and booster vaccination, respectively), the responses detected in the primary effector samples were much higher than those obtained in the secondary effector samples, and this difference was highly significant.

### Magnitude of the humoral/antibody response after primary and booster vaccination

**Figure 4:**
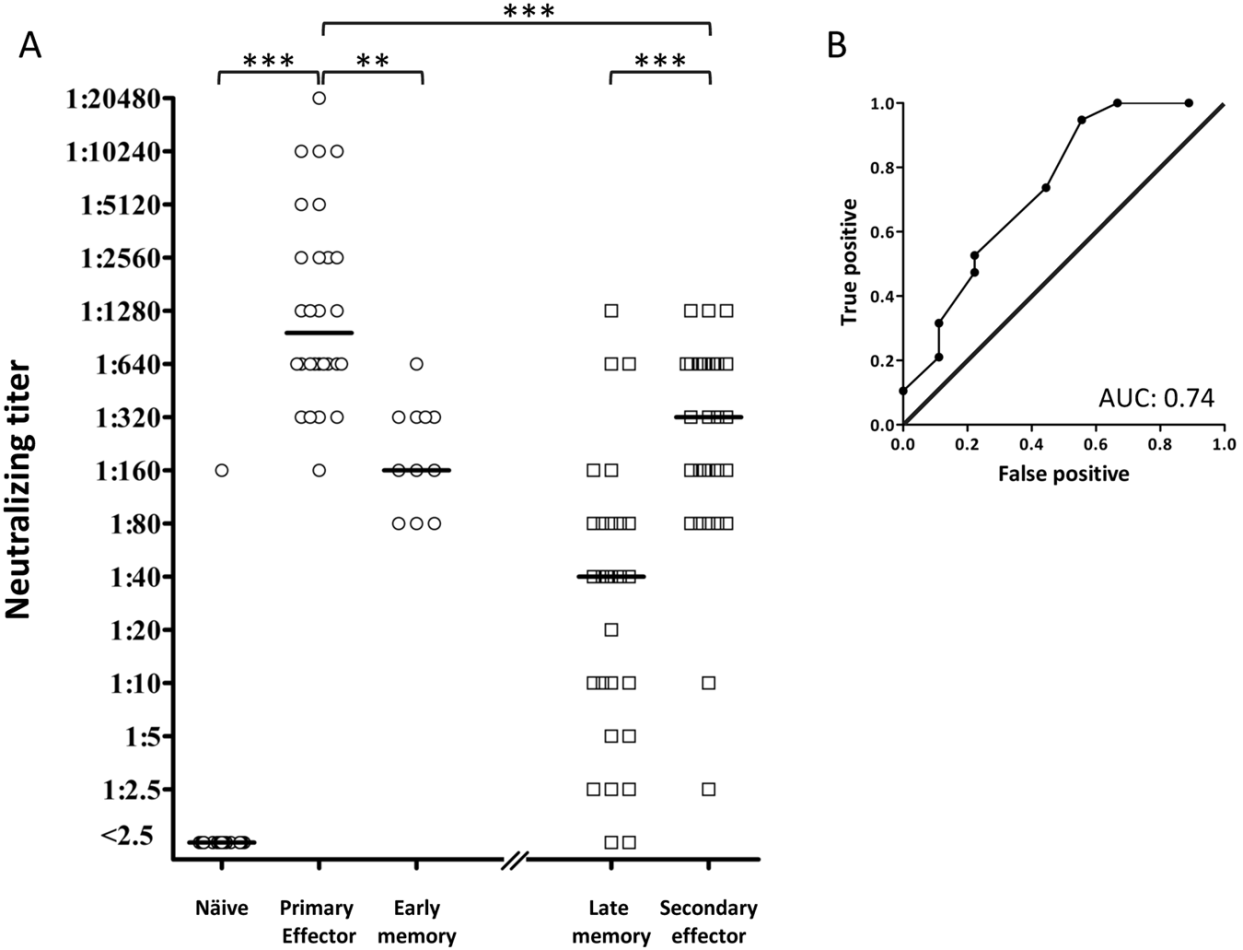
Antibody responses after primary and booster YF-17D vaccination. (**A**) Using a Plaque Reduction Neutralization Titre (PRNT) assay^27^, YFV specific neutralizing antibodies were assessed in sera from primary vaccinated donors (n = 28) before (“naïve”, n = 28), 12–22 days post-vaccination (“primary effector” n = 28), approximately 2 years post vaccination (“early memory”, n = 11); and from booster vaccinated donors before (“late memory”, n = 28) and after (“secondary effector”, n = 28) vaccination. The PRNT_50_ titre was defined as the reciprocal of the last serum dilution, which caused a 50% reduction in the number of plaques. The median is indicated. Mann Whitney U test was used to determine the significance of the difference between the indicated groups (***p < 0.0001; **p = 0.006). (**B**) A “Receiver Operating Characteristics” (ROC) analysis was performed to evaluate whether a high neutralizing antibody titre in the booster vaccinated donors prior to vaccination correlated with a reduced vaccination-induced titre increase (the latter classically defined as being >4-fold). True-positive rate is depicted against the false-positive rate at various thresholds of pre-booster vaccination titres. The area under this ROC curve (AUC) is 0.74 (p < 0.046).

Antibodies are the main effector mechanism in clearing Yellow Fever infection^27, 35, 36^. To examine the antibody responses in primary and booster vaccinated donors we used a plaque-reduction neutralizing test (PRNT) as a functional readout of the capacity of the vaccine to generate protective humoral immune responses. This PRNT assay was performed on pre- and post-vaccination sera from 28 primary and 28 booster vaccinated donors. Furthermore, memory samples from 11 of the primary vaccinated donors were collected approximately 2 years after the primary vaccination. The result of the neutralization test is seen in Fig. 4A. None of the naïve donors, except one, had detectable antibodies. In contrast, all primary vaccinated donors generated neutralizing antibodies (a median titre of 1,280 (range from 160 to 20,480)). Two years after the primary vaccination, the neutralizing titre had fallen significantly in all 11 donors examined (a median titre of 160 (range from 80 to 640)). The neutralizing titres had fallen further prior to booster vaccination (a median titre of 20 (range from <2.5 to 1,280)). Five of these 28 late memory stage donors, who had been primary vaccinated 26, 23, 11, 11, and 9 year before, had titres below 10. The remaining 23 late memory donors had titres above 10 including donors that had been primary vaccinated 36 and 25 years ago, who had titre of 80 and 640, respectively. The booster vaccination led to a significant increase in neutralizing titre (a median of 1:160 (range from 1:80 to 1:1,280)); 21 of the 28 booster vaccinated (75%) experienced a four-fold, or higher, increase in neutralizing titres, which conventionally is defined as being significant. Five experienced a non-significant two-fold titre increase, and the two remaining boosted donors had unaltered neutralizing titres of 2.5 and 10, respectively.

### The effect of pre-existing antibodies on humoral immune responses following booster vaccinations of human vaccinees

Thus, whether judged by evaluation of CD8^+^ T cell, CD4^+^ T cell or humoral immune responses, the same conclusion was reached: that secondary effector responses against YF-17D vaccination are significantly lower than primary effector responses. We speculated that these reduced secondary effector responses could be caused by antibody-dependent neutralization and a diminished virus load leading to a reduced stimulation of the immune system^35^. To address this question, we examined whether high “pre-boost” titres correlated with reduced immune response to booster vaccination. Classically, >4-fold titre increase between paired samples is considered a strong indication of recent infection. Comparing “pre-boost” vs. “post-boost” neutralization titres, we observed >4-fold titre increase in 19 of 28 booster vaccinees. Examining whether a low “pre-boost” neutralizing titre correlated with >4-fold “post-boost” titre increase, we performed a “Receiver Operating Characteristics” (ROC) analysis where the true-positive rate is depicted against the false-positive rate at various thresholds of “pre-boost” titres. The area under this ROC curve, the “Area Under the Curve” (AUC), is 0.74 (where an AUC of 1.00 defines a perfect relationship, and an AUC of 0.500 defines a random relationship) (Fig. 4B). This AUC is significant at the 5% level supporting the hypothesis that pre-existing antibodies inhibit the humoral immune response following a booster vaccination.

### The effect of pre-existing antibodies on cellular immune responses following booster vaccinations in an animal model

**Figure 5:**
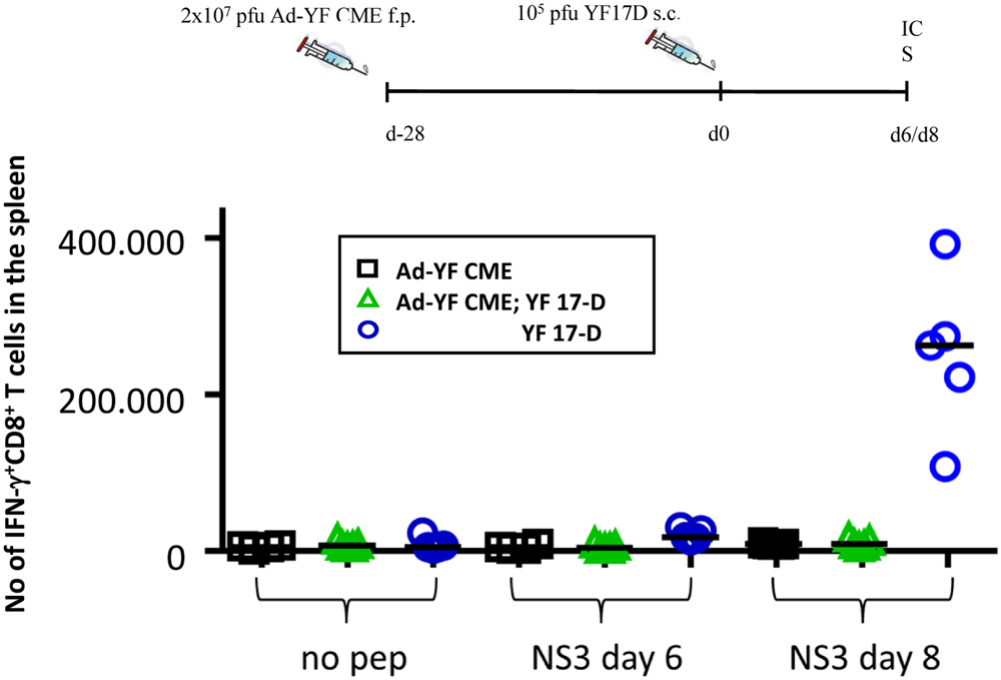
Pre-existing immunity to the YFV structural proteins (C,M,E) inhibits the CD8^+^ T cells response induced by YF-17D vaccination in mice. C57BL/6 (H-2b) mice were either untreated or vaccinated with an experimental adenoviral vectored vaccine, Ad-YF CME, directed against the structural proteins, core, membrane and envelope (C, M, E). Twenty-eight days later, all unvaccinated mice and half the vaccinated mice were s.c. immunized with the YF-17D vaccine, and the CD8+ T-cell response to the non-structural protein NS3 was determined. Following *ex vivo* peptide stimulation of splenocytes with NS3_268-275_, the number of CD44+IFN-γ+CD8+ T cells were determined 6 and 8 days post YFV infection using an intracellular cytokine staining (ICS); non-stimulated (no pep) cells served as controls. Symbols denote individual animals.

Unfortunately, we were unable to demonstrate a significant correlation between the presence of pre-existing antibody and reduced cellular immune responses following booster vaccination in humans. To perform this analysis in a better controlled system, we analysed CD8^+^ T cell responses induced by YFV vaccination in mice that had, or had not, been previously immunized with a recombinant Adenoviral vectored vaccine encoding the structural YF-17D proteins, core, membrane and envelope, denoted Ad-YF CME. This vector has been shown to induce neutralizing antibody titres detectable from day 7 to at least day 60 post-vaccination. In mice, the whole attenuated YF-17D vaccine induces a CD8^+^ T cells response, which peaks at day 7 after infection and is specific for a dominant viral epitope present in the NS3 protein; this latter NS3-specific, CD8^+^ T cell response is not induced by the Ad-YF CME vaccine^32^. C57BL/6 mice, which had, or had not, been immunized with 2 * 10^7^ pfu of the Ad-YF CME vaccine in the footpads (f.p.) 28 days earlier, were subcutaneously (s.c.) infected with 10^5^ pfu of the YF-17D vaccine. Subsequently, the splenic CD8^+^ T cell responses were analysed by IFN-γ-specific intracellular cytokine staining (ICS) at day 6 and 8 after the YF-17D vaccination. A group of mice, which had only received the Ad-YF CME vaccination, was included for analysis of the memory CD8^+^ T cells response at the time of re-infection with the YF-17D vaccine. We found that NS3-specific CD8^+^ T cells could be detected in all the YF-17D vaccinated mice at day 8, but not day 6, after vaccination; strikingly, mice that had been immunized with the Ad-YF CME vaccine 28 days prior to YF-17D vaccination failed to generate NS3-specific CD8^+^ T cells (Fig. 5). These data support the notion that pre-existing YFV-specific antibodies can inhibit T cell responses induced by YF-17D vaccination.

## Discussion

Until recently, the World Health Organization (WHO) has recommended that Yellow Fever “booster” vaccination of individuals at risk should be given ten years after past vaccination. In 2013, however, the WHO “Strategic Advisory Group of Experts on Immunization” (SAGE) removed this recommendation for healthy individuals since it considered a single dose of yellow fever vaccine sufficient to confer life-long protective immunity against the disease^9, 37^. The SAGE recommendation was adopted by the World Health Assembly in 2014, and entered into force legally (i.e. be removed from the International Health Regulations (IHR)) in June 2016. Similarly, the US Advisory Committee on Immunization Practices (ACIP) has voted that a single primary dose of yellow fever vaccine provides long-lasting protection and is adequate for most travelers^38^. It should be noted that the decision to discontinue to YFV booster vaccinations has been the subject of some discussion^37, 39, 40^.

Unfortunately, what constitutes sufficient protection against Yellow Fever virus infection is not fully understood, and there is no accepted correlate of immune protection against YFV. Antibodies are generally considered to be the dominant protective mechanism of vaccines^14, 15, 35, 36, 41^ and have conventionally been viewed as the most appropriate correlate of immune protection. In this context, a single immunization generates neutralizing antibody titres, which may be detected in some vaccinees within 6 days, in 80–90% of vaccinees within 10 days, and in more than 99% within 28 days after vaccination^1, 2, 9, 10, 13, 15, 41^. Neutralizing antibody titres may persist for up to 45 to 60 years after vaccination^13–15, 42, 43^. Here, we found an average neutralizing (PRNT) antibody titre of 1:1280 (range 1:160 to 1:20,480) in primary vaccinees tested 9–40 days after vaccination (Fig. 4A). That neutralizing antibodies is the dominant protective immune mechanism induced by YF-17D vaccination is corroborated by animal experiments involving either passive transfers of antibodies^41^ or genetically introduced immune deficiencies^27^.

While recognizing that neutralizing antibodies are associated with protection, SAGE also noted that “*cellular immunity and innate immunity contribute to the initial immune response and sustaining the immune memory to yellow fever vaccination*” and suggested “*that a lack of detectable neutralizing antibodies may not mean a lack of protection against yellow fever viral infection among yellow fever vaccine recipients*”. A priori, one would expect that many other arms of the immune system, innate as well as adaptive, are involved in the immune response induced by a primary YF-17D vaccination, and that all of these immune responses contribute to the superior efficiency of the YF-17D vaccines^16, 18, 20–23, 26, 29, 34, 44^. Already Theiler observed that vaccinated animals were protected before neutralizing antibody titres could be detected^43^; something, which in hindsight could implicate additional protective immune mechanisms^41^. However, it is difficult to assess the effect of protective non-antibody mechanisms in the presence of a dominant antibody activity. Recently, we used genetically engineered mouse models to circumvent the obscuring effects of antibodies and address the effects of the remaining immune mechanism. In YFV vaccinated mice, we demonstrated an early (around day 5–7) and protective influx of specific CD8^+^ T cells, and CD8 T cell depletion resulted in increased organ viral load; results that to the best of our knowledge are the first demonstration of the protective effect against Yellow Fever virus of CD8^+^ T cells in isolation^27^.

Others have also suggested a role for CD8^+^ T cells. Miller *et al*. identified and used an activated CD8^+^ T cell phenotype, defined as being CD38^+^, HLA-DR^+^, KI-67^+^ and Bcl-2^low^, to analyse human CD8^+^ T cell activation and demonstrated that the human immune response to YF-17D vaccination involves a brisk expansion and activation of CD8^+^ T cells^26^. Here, using a simplified CD38^+^/HLA-DR^+^ phenotype to analyse the immune responses of 209 of 210 primary YF-17D vaccinees, we observed a rapid increase in the percentage of activated CD8^+^ T cells following YF-17D vaccination making this response detectable already around day 10–11, reaching a highly significantly peak plateau from around day 12 to 22 where an average of 5% (range 1 to 20%) of all CD8^+^ T cells were activated, following which they rapidly subsided during the fourth week of the response (Fig. 1A). Using the same simplified CD38^+^/ HLA-DR^+^ phenotype, we observed a similar, albeit less prominent, activation kinetics of CD4^+^ T cells (Fig. 3A). Implicitly, both CD8^+^ and CD4^+^ T cells must go through expansion since they a priori have emanated from T cell populations with very few YFV-specific CD8^+^ and CD4^+^ T cells.

A more direct analysis of CD8+ T cell activation and expansion, would be to use HLA class I tetramers to identify CD8^+^ T cells at the single cell level and then co-stain these for the CD38 and HLA-DR activations markers. Initially, we used an NS4B_214-222_/HLA-A*02:01 tetramer, which represents a particularly strong and immunodominant YF-17D response. Our cohort of YF-17D vaccinees included 93 primary vaccinated HLA-A*02:01 donors allowing us to address the kinetics of activation. From the earliest time point observed (9 days post vaccination) until day 16, virtually all NS4B_214-222_/HLA-A*02:01 specific CD8^+^ T cells had an activated phenotype. At that time, a contraction and de-activation phase started, where the fraction of activated, NS4B_214-222_/HLA-A*02:01 specific CD8^+^ T cells began to drop reaching about 50% activated specific CD8+ T cells around day 22 and returning to low levels about day 30 (Fig. 1B and D). As shown here (and in unpublished results, A Stryhn), this epitope is recognized in every single YF-17D vaccinated, HLA-A*02:01 donor that we have examined; the frequencies of T cells responding *ex vivo* is unusually high (in some cases approaching 11%); and, in contrast to other CD8+ T cell responses we have examined, this specificity can be expanded *in vitro* in many naïve, HLA-A*02:01 positive, donors. The latter is in agreement with Blom *et al*.^29^, who observed that the NS4B_214-222_ peptide could stimulate a small, but detectable, IFN-γ production in three of 9 naïve HLA-A*02:01 individuals. It has been suggested that these responses are the result of pre-existing, cross-reaction towards other pathogens^45^.

Thus, we were concerned that the unusually strong response of the NS4B_214-222_/HLA-A*02:01 specificity might not reflect primary response kinetics in general. To get a more representative picture of the activation kinetics of CD8^+^ T cells after YF-17D vaccination, we expanded the activation analysis of tetramer-specific cells to 11 additional YF-17D-derived epitopes, restricted to 11 HLA-I elements (see tetramer section of Materials and Methods). Compared to the NS4B_214-222_/HLA-A*02:01 epitope, these other epitopes are generally less dominant, less frequent and do not show the same *in vitro* expansion and activation potential in naïve individuals. However, comparing the NS4B_214-222_/HLA-A*02:01 response with the 11 other responses, similar kinetics were observed. A priori, one should expect a stronger and more frequent CD8^+^ T cell response to clear an infection faster than a weaker and less frequent response, and that such a difference could be reflected in the deactivation kinetics, however, our results seems to indicated that this is not the case. Rather than being related to the resolution of the infection, the deactivation kinetics could be related to the gradual effector-to-memory transition of pre-programmed CD8^+^ T cells^21, 26^. In two donors, we consecutively sampled and analysed the expansion and activation of YF-17D-specific CD8^+^ T cells over a longer time period using relevant peptide-HLA-I tetramers (Fig. 1D). Early in the response and up to about day 20 day, all the tetramer-specific primary effector CD8^+^ T cells were of the activated phenotype. Thereafter, the population started to lose activation markers and diminish, however, even at day 450 and 960, a low, but detectable, number of tetramer-specific late memory CD8^+^ T cells could be observed. Thus, irrespective of which tetramer-specificities we monitored, the kinetics were in good agreement with the general activation kinetics observed in Fig. 1A, and with the kinetics reported by Akondy *et al*.^21^.

Strikingly, we found that the magnitudes of secondary (booster) responses induced by YF-17D were much reduced compared to primary responses. This reduction was observed for all parameters measured here: neutralizing antibody titres; frequency of total activated CD4+ and CD8+ T cells, and of YFV-specific CD8+ T cells; and functional responses of YFV-specific CD4+ and CD8+ T cells. Wisseman *et al*. revaccinated eleven volunteers with YF-D17 about one year after primary vaccination and observed a reduced secondary neutralizing antibody response compared to the primary response^46^. In contrast, Reinhardt *et al*. revaccinated five volunteers more than 10 years after the primary YF-17D vaccination and observed a higher neutralizing antibody response in these revaccinated volunteers than in twelve primary vaccinated volunteers^41^. Revaccinating with the attenuated measles vaccine, Christenson *et al*. showed that secondary antibody responses (as studied by ELISA, >300 subjects) yielded significantly lower titres than primary vaccination, and that prior natural measles infection completely abrogated secondary antibody responses^47^. Other studies have compared the magnitude of the secondary humoral response to either the attenuated yellow fever (YF-17D) or measles vaccines as a function of the pre-existing (residual) antibody titres, and found that the higher residual antibody titre was, the lower the resulting secondary antibody response became^14, 48, 49^. Our analysis supports this inverse relationship between the magnitudes of the primary and secondary immune responses.

In animal models, several studies support the concept that pre-existing immunity, not only antibodies, but also T cells, may reduce the host response^27, 32^. Overall, our results are in agreement with the consensus view expressed by Siegrist that “*secondary responses to live attenuated viral vaccines are minimal, as pre-existing antibodies mostly neutralize the vaccine load prior to its in vivo replication*”^35^. In fact, one could argue that any kind of primary induced, acquired immune response, which could reduce the replication of a vaccine vector at the time of a revaccination challenge, would reduce the resulting secondary immune response^50^; and that any kind of secondary response could potentially be used to monitor this effect. In the context of the latter, Akondy *et al*. has recently demonstrated that the initial YF-17D load determines the magnitude of the CD8+ T cell response^51^; something that would suggest that the CD8+ T cell response could be used to monitor the effect of pre-existing immunity. Indeed, using a mouse model of Yellow Fever infection and a non-replicating adenoviral vectored vaccine, we here demonstrate that pre-existing immunity inhibit vaccine induced specific CD8+ T cell responses, and using human volunteers and the attenuated YF-17D vaccine, we further demonstrate that secondary CD8+ T cell responses are significantly reduced compared to primary responses. Besides the implications for YFV vaccination, our findings also raise a cautionary note regarding the practical use of YF-17D as a vaccine vector. Depending on the YF genes remaining after such genetic manipulations, efficient priming of immune responses directed against the heterologous components inserted into such a chimeric YF-17D-derived vaccine may be severely compromised by any pre-existing immunity directed against the YF-17D vector itself.

The use of YFV “booster” vaccinations was recently removed from the International Health Regulations. This was partly founded in the long-lived, probably life-long, immunity against Yellow Fever infection afforded by a single primary YFV immunization, partly in limited supplies of the YF-17D vaccine. Whilst it is true that high titres of neutralizing antibodies can be found decades after primary vaccination, this is not necessarily true for all primary vaccinated. About 25% of our volunteers, who were about to receive a booster vaccination 10 years, or more, after primary infection, had a PRNT titre of less than 1:5, and all, but one, responded with a significant increase in titres suggesting that the primary immune response was waning leading to a suboptimal immunity towards YFV thus allowing a booster dose of attenuated virus to infect its target cells and replicate. One could therefore argue that these individuals would benefit from a booster vaccination. However, in a situation with a limited supply of the vaccine and where new outbreaks occur regularly, one could also argue that immunizing naïve individuals at risk is a priority and a more efficient use of available vaccine supplies^9^.

## Electronic supplementary material

Supplementary figures
