# Supplementary material for: Adaptive immune responses to booster vaccination against yellow fever virus are much reduced compared to those after primary vaccination
Source: Sci Rep. 2017 Apr 6;7:662. doi: 10.1038/s41598-017-00798-1 (PMC5429613; doi:10.1038/s41598-017-00798-1)
Supplement: Supplementary file 1 — Supplementary figures [file 41598_2017_798_MOESM1_ESM.pdf]

Adaptive immune responses to booster vaccination against yellow fever virus are much reduced compared to those after primary vaccination

Michael Kongsgaard[1], Maria R. Bassi[1], Michael Rasmussen[1], Karsten Skjødt[2], Søren Thybo[3], Mette Gabriel[4], Morten Bagge Hansen[5], Jan Pravsgaard Christensen[1], Allan Randrup Thomsen[1], Soren Buus[1*] and Anette Stryhn[1*].

1 Department of Immunology and Microbiology, University of Copenhagen, Copenhagen, Denmark.
2 Department of Cancer and Inflammation, Institute for Molecular Medicine, University of Southern Denmark, Odense, Denmark.
3 Department of Infectious Diseases, Copenhagen University Hospital, Copenhagen, Denmark
4 Medical Office, Copenhagen, Denmark
5 Department of Clinical Immunology, Copenhagen University Hospital, Copenhagen, Denmark

Supplementary figures

Supplementary figure S1: Comparison of HLA-DR+/CD38+ and Ki67+/Bcl2- detection of activated CD8+ T cells after YF-17D vaccination.
PBMCs from 190 primary YFV vaccinated donors were extracellular and intracellular stained with fluorochrome-conjugated antibodies specific for CD3, CD8, CD38, and HLA-DR or CD3, CD8, Ki67, and Bcl2, respectively. For each donor the frequency of $CD38^+$ HLA-DR^+ $CD8^+$ T cells are plotted against the frequency of $Ki67^+$ $Bcl2^-$ $CD8^+$ T cells, respectively. The line indicates y = x.

Supplementary figure S2: A representative flow cytometric analysis of T cell activation.
PBMCs from a primary vaccinated donor obtained before (pre) and after (post) YF-17D vaccination were stained *ex vivo* with CD3, CD8, CD4, CD38 and HLA-DR specific antibodies. The two left-hand panels show $CD8^+$ T cells obtained before and after YF-17D vaccination. The population gated in red represents the activated $CD38^+$ HLA-DR^+ $CD8^+$ $CD3^+$ T cells. The two right-hand panels show $CD4^+$ T cells obtained before and after YF-17D vaccination. The population gated in green represents the activated $CD38^+$ HLA-DR^+ $CD4^+$ $CD3^+$ T cells.

Supplementary figure S3: A representative HLA-A02:01/$NS4B_{214-222}$ tetramer-based analysis of specific $CD8^+$ T cells before and after YF-17D vaccination.
PBMCs from a HLA-A*02:01 positive donor obtained before (pre) and after (after) primary YF-17D vaccination were stained *ex vivo* with a $NS4B_{214-222}$/HLA-A*02:01 tetramer, and with anti-CD3 and -CD8 specific antibodies. The percentage of $tetramer^+$, $CD8^+$ T cells is indicated. The specificity of the tetramer staining is indicated by the increased staining after vaccination as well as the lacking binding to irrelevant cells such as the $CD8^-$ (i.e. $CD4^+$) T cells.

Supplementary figure S4: Representative staining of specific CD8+ T cells using 11 additional tetramers.
PBMCs from HLA class I-typed primary vaccinated donors were obtained after YF-17D vaccination and stained *ex vivo* with the indicated tetramers, and with anti-CD3 and -CD8 specific antibodies. Staining of a well-defined population of CD8+ T cells, but not of CD4+ T cells, illustrates the specificity of the tetramers. In contrast, the tetramer background staining is a dispersed staining of <0.009%.

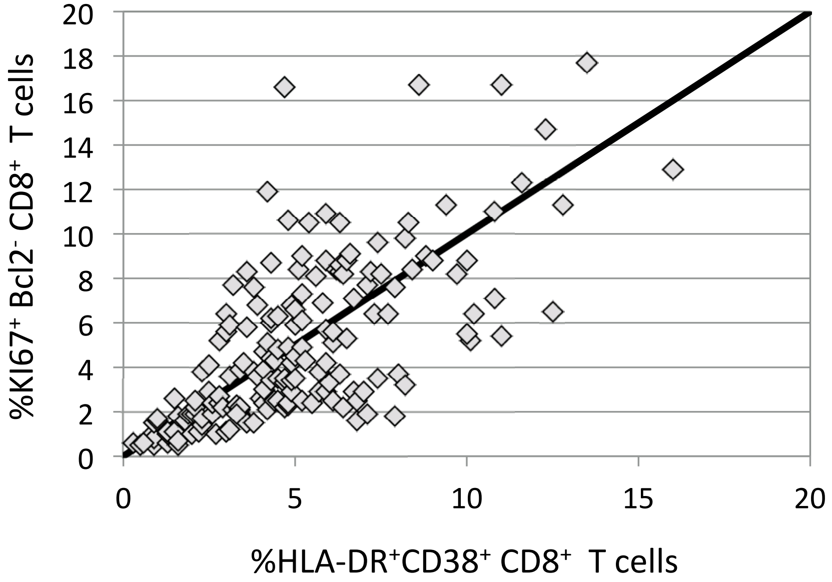

Kongsgaard et al, Supplementary Figure 1

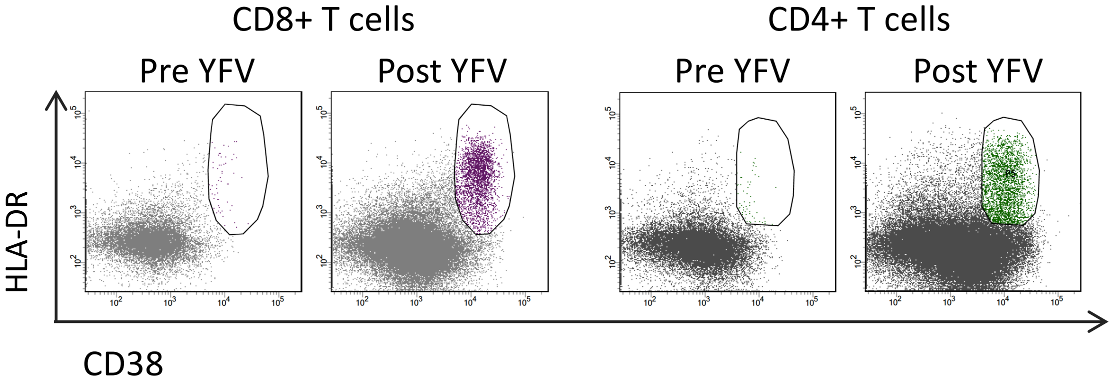

Kongsgaard et al, Supplementary Figure 2

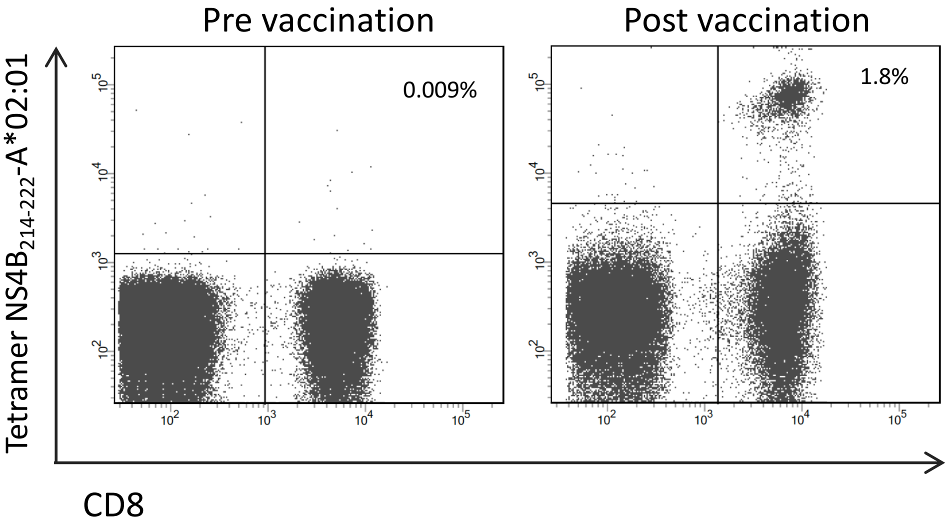

Kongsgaard et al, Supplementary Figure 3

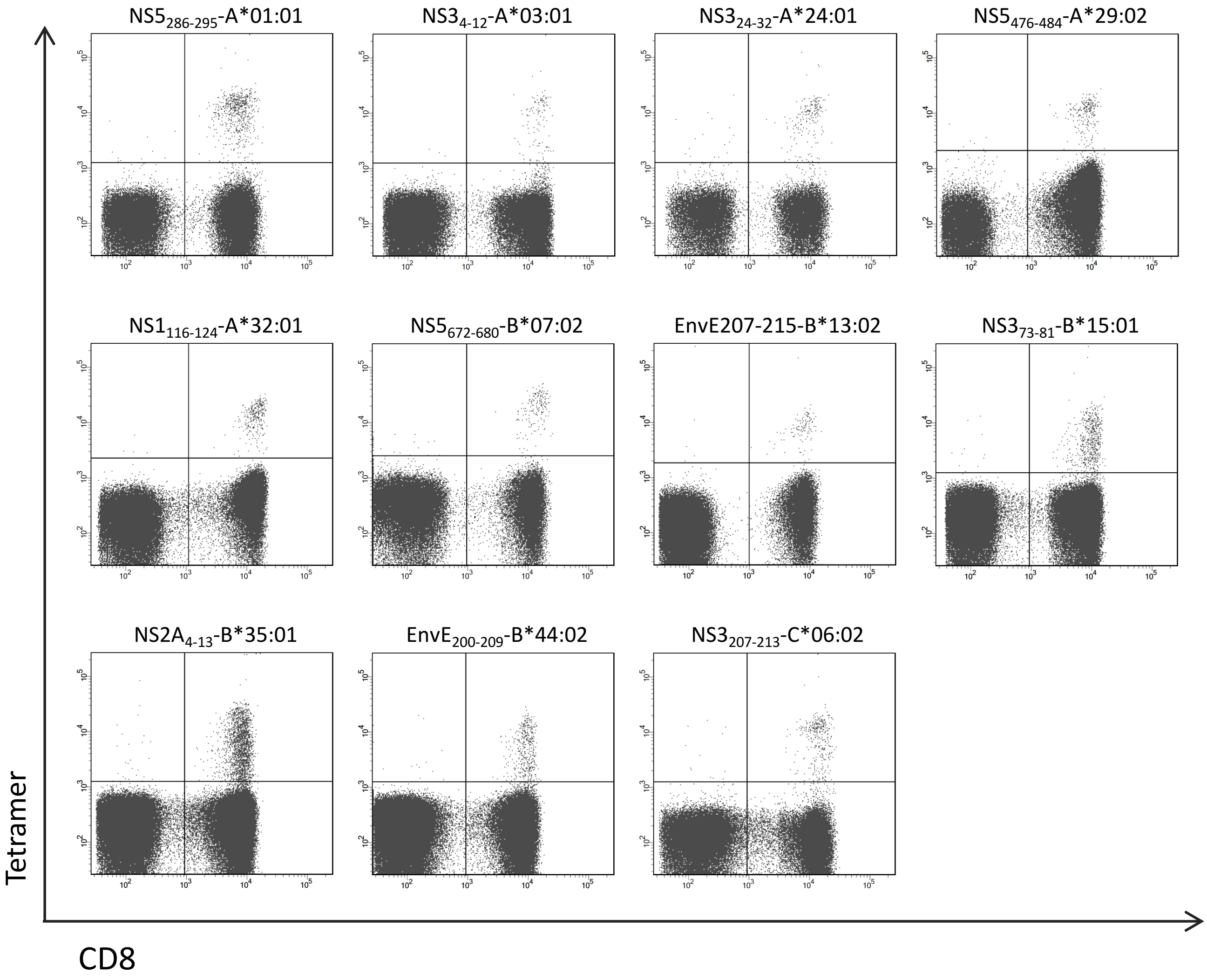

Kongsgaard et al, Supplementary Figure 4
